# A MATLAB-based program for three-dimensional quantitative analysis of micronuclei reveals that neuroinflammation induces micronuclei formation in the brain

**DOI:** 10.1038/s41598-021-97640-6

**Published:** 2021-09-15

**Authors:** Sarasa Yano, Kaito Akiyama, Rio Tsuchiya, Hikari Kubotani, Tomoki Chiba, Takeshi Nagata, Fuminori Tsuruta

**Affiliations:** 1 Graduate School of Life and Environmental Sciences, University of Tsukuba, Tsukuba, Japan; 2 College of Biological Sciences, School of Life and Environmental Sciences, University of Tsukuba, Tsukuba, Japan; 3 Master’s and Doctoral Program in Biology, Faculty of Life and Environmental Sciences, University of Tsukuba, Tsukuba, Japan; 4 Master’s and Doctoral Program in Neuroscience, Graduate School of Comprehensive Human Sciences, University of Tsukuba, Tsukuba, Japan; 5 Ph.D. Program in Human Biology, School of Integrative and Global Majors, University of Tsukuba, Tsukuba, Japan; 6 Ph.D. Program in Humanics, School of Integrative and Global Majors, University of Tsukuba, 1-1-1 Tennodai, Tsukuba, Ibaraki 305-8577 Japan; 7 Information and Communication Research Division, Mizuho Research and Technologies, Ltd., Inc., 2-3 Nishiki-cho, Kanda, Chiyoda-ku, Tokyo, 101-8443 Japan

**Keywords:** Neuroimmunology, Cellular neuroscience, Nucleus

## Abstract

The micronucleus is known to be a biomarker for genomic instability, which is a hallmark of tumors and aging. Normally, micronuclei are produced by segregation errors and mechanical stresses arising from dividing or migrating cells, leading to activation of the innate immune response pathway. Although micronuclei often emerge in damaged tissues, the quantitative procedure for analyzing micronuclei accurately has been problematic. Here, we introduce a novel MATLAB-based program for quantifying micronuclei (CAMDi: calculating automatic micronuclei distinction) in vitro and in vivo. CAMDi is adaptable to various experimental imaging techniques and is useful for obtaining reproducible data. CAMDi enables us to measure the accurate size of micronuclei from the three-dimensional images. Using CAMDi, we revealed a novel link between the emergence of micronuclei and neuroinflammation. We found that inflammatory stimulation does not increase the number of micronuclei in primary neurons. On the other hand, the administration of lipopolysaccharide into mice slightly increases micronuclei formation in neurons of the hippocampus region. These findings demonstrate that neuronal micronuclei formations are induced by an inflammatory response in a non-cell-autonomous manner. We provide a novel tool, CAMDi, to quantify micronuclei and demonstrate that neuronal micronuclei are produced not only by the cell-autonomous process but also by the intercellular communication associated with neuroinflammation in vivo.

## Introduction

The micronucleus is a small extranuclear structure surrounded by a nuclear envelope that contains chromatin and nuclear proteins. Historically, the micronuclei were identified as small basophilic nuclear remnants in the erythrocytes over one hundred years ago^1^. In the middle of the twentieth century, these small nuclei were also identified in other tissues^2^. Currently, these small nuclei are generally referred to as micronuclei^3^. Micronuclei are produced as a result of various genotoxic stressors and act as biomarkers of DNA damage. Micronuclei are also known to increases the risk of genomic instability and tumor malignancy. As such, the analysis of micronuclei is beneficial to our understanding of the genomic conditions of each cell and tissue^4–6^.

To date, it has been considered that the micronuclei are efficiently produced by inhibiting cytokinesis. For example, stimulation with cytokinesis-related inhibitors, such as paclitaxel, promotes abnormal nuclei formations in vitro and in vivo^7,8^. These reagents frequently induce the mis-segregation of sister chromatids by inhibiting the fine chromatid orientation and abnormal tension at the kinetochore, which is the main cause of micronuclei formations. Other stimulation, such as DNA damage, is associated with micronuclei formation. γ-ray irradiation in vitro is positively correlated with micronuclei formations^9^. It is well known that γ-ray irradiation triggers a double-strand break and DNA fragmentation. If the level of DNA damage exceeds the repair capacity, the probability of micronuclei formation is markedly increased via aberrant homologous recombination and non-homologous end-joining machinery. Moreover, treatment with genotoxic reagents, such as etoposide, methanesulfonate, and bleomycin, results in micronuclei formation^10^. Since these reagents often hamper DNA repair and replication, stimulating cells with these reagents damages chromosomes. When these damaged cells that are stimulated by genotoxins are divided into daughter cells, chromosome segregation errors frequently occur and produce aneuploidy and chromosome fragmentation, resulting in abnormal nuclear formation. Thus, micronuclei are produced by various genotoxic stimulations and chromosomal instability. Moving cells also incur marked mechanical stress when they migrate through narrow spaces^11^. During this migration, cells change its shape dramatically and undergo significant mechanical stress. These mechanical stresses elicit abnormal changes in the nuclear structure, promoting nuclear deformation. Indeed, previous studies have reported that the nuclear shape is deformed when cancer cells penetrate a tight interstitial region. Although the damaged nuclei are repaired by the endosomal sorting complexes required for transport (ESCRT) III complex after rupturing the nuclear envelope, some nuclear components leak into the cytoplasm^12,13^. Thus, the nuclear deformation and envelope rupture elicited by physical stress frequently causes micronuclear production. Since these micronuclei are non-essential components in cells, they are usually cleared by the autophagy pathway to maintain an intracellular environment. The autophagy pathway is tightly controlled by ATG family proteins. Previously, it was demonstrated that the LC3/Atg8 and Atg39 regulate the degradation of the nucleus and its lamina by the autophagic pathway in mammalian cells and yeast^14,15^. In fact, the LC3/Atg8, which is a regulator for inducing autophagosomes, colocalized on micronuclei in cancer cells^16^, indicating that autophagic machinery is associated with micronuclei clearance.

In pathological conditions such as cancer, micronuclei are frequently observed in cancer cells and are utilized as biomarkers for tumorigenesis. The nuclear architecture of cancer cells exhibits unusual shape, size, and chromatin texture because the nuclear envelope in cancer cells is fragile and their morphology is easy to deform^17^. These observations call into question the biological relevance between micronuclei and tumorigenesis in vivo. Recently, it has been reported that micronuclei enhance the pro-inflammatory pathways and metastasis in cancer cells^18,19^. In cancer cells, the emergence of micronuclei promotes the cellular invasion ability by sustaining a cell-autonomous process such as the cyclic GMP–AMP synthase (cGAS)–stimulator of interferon genes (STING) pathway. Suppression of micronuclei formation and the cGAS-STING pathway significantly delays metastasis even in the aneuploid cells, indicating that micronuclei increase the malignant transformation of the cancer cells. Moreover, micronuclei are associated with chromothripsis^20^. Chromothripsis is a catastrophic event that causes cellular transformation by genomic rearrangements. Micronuclei formation may trigger chromothripsis, leading to carcinogenesis or the elimination of abnormal chromosomes. Research on micronuclei in cancer cells has made marked progress. Micronuclei can also be observed in other tissues, including the brain. For instance, micronucleus is associated with neurodegenerative disorders and acts as a biomarker for them^21^. Since excess inflammation, which is frequently observed in the neurodegenerative brain, causes DNA damage in tumor cells, neuroinflammation could be a potential trigger that induces micronuclei formation in the brain^22^.

Several studies have reported approaches to quantify micronuclei in vitro and in vivo. The most common approach involves detecting the small nuclei by nuclear staining and imaging manually or automatically^9,23–26^. These standard approaches are employed to count the number of small nuclei by setting the threshold, such as the nuclear size. This approach is used to obtain data effectively and for general use. Quantification in conjunction with high-throughput screening and machine learning is powerful for analyzing the genomic condition in vitro^25,26^. It is technically difficult, however, to obtain precise data on the micronucleus in tissues due to stereoscopic challenges (see the Results sections). It is also difficult to distinguish the micronuclei from each nucleus when the nuclei are tightly packed in a limited area. Therefore, the development of another application tool to quantify micronuclei precisely has been awaited.

In this study, we introduce a MATLAB-based software, CAMDi (calculating automatic micronuclei distinction). CAMDi is a useful program that quantifies the micronuclei precisely based on Z-stack position information and enable us to acquire the accurate size of micronuclei. CAMDi can quantify micronuclei in both culture cells and thick tissue sections. Moreover, the CAMDi can distinguish the subcellular localization of micronuclei via triple staining and ellipse fitting functions even in dense conditions. Using CAMDi, we investigate the link between micronuclei formation and neuroinflammation. Although treatment with inflammatory cytokines induces neuroinflammation but has little effect on producing micronuclei in primary neurons, the administration of lipopolysaccharide (LPS) significantly induces micronuclei formation in the hippocampus region. These observations suggest that neuroinflammation promotes micronuclei formation.

## Results

### Micronuclei emerge in various tissues of adult mice

**Figure 1:**
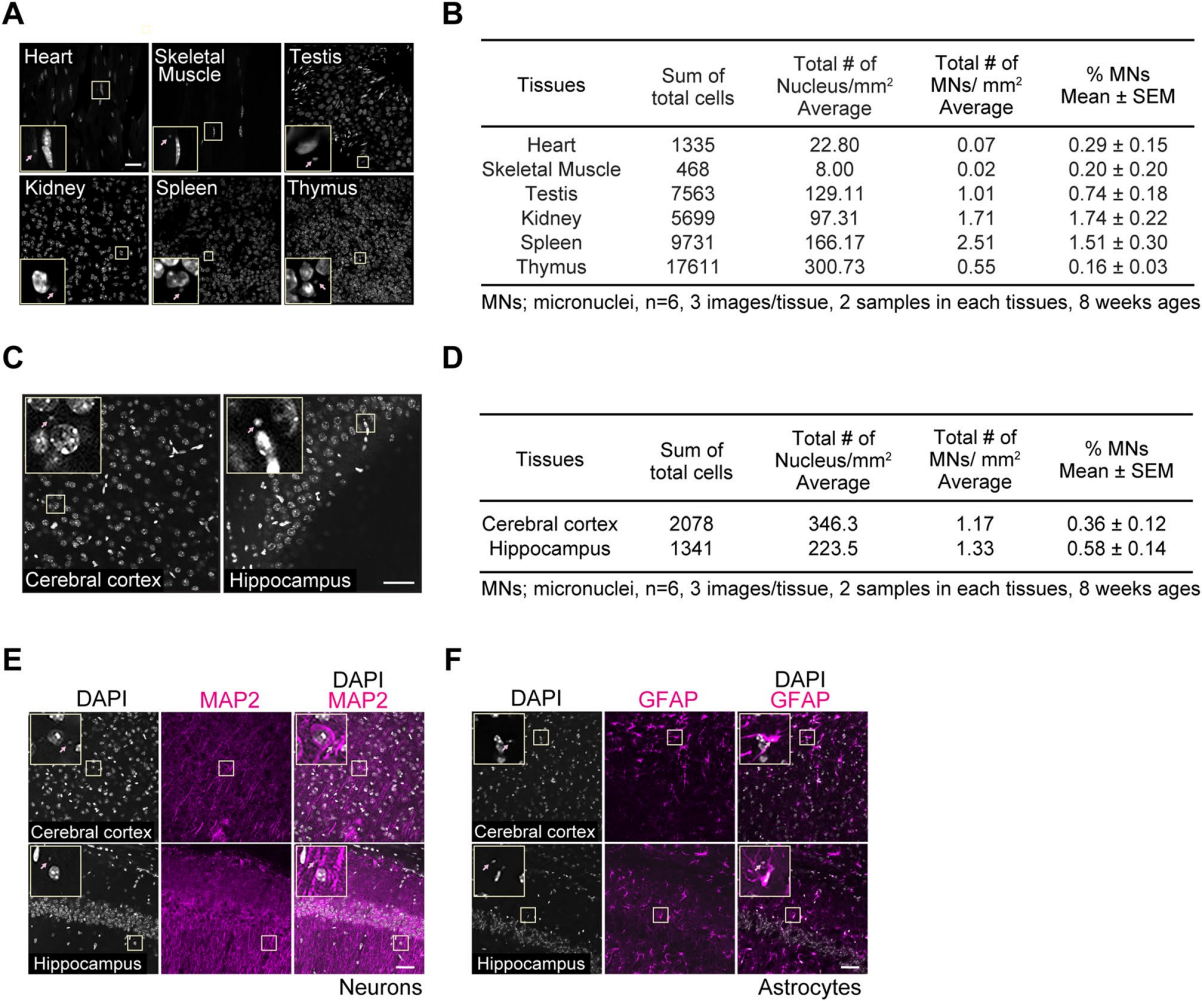
Micronuclei emerge in various tissues of adult mice. (**A**) Nuclei in each tissue (heart, skeletal muscle, testis, kidney, spleen, thymus) from 2-month-old mice were stained with Hoechst 33,342. Insets show an enlargement of the micronuclei in the boxed area. Arrows indicate micronuclei. Scale bar: 50 μm. (**B**) Table shows the quantification of micronuclei in Fig. 1A by human observers. The small nucleus-like structure, which is approximately less than 2.0 μm, was counted as a micronucleus. The three images were obtained from the tissue sections prepared from a single mouse. The other three images were obtained from an alternate mouse (a total of 6 images from 2 mice brains). (**C**) Nuclei in the brain (cerebral cortex and hippocampus) from 2-month-old mice were stained with DAPI. Insets show an enlargement of the micronuclei in the boxed area. Arrows indicate micronuclei. Scale bar: 50 μm. (**D**) Table shows the quantification of micronuclei in Fig. 1C by human observers. The small nucleus-like structure, which is approximately less than 2.0 μm, was counted as a micronucleus. The three images were obtained from the tissue sections prepared from a single mouse. The other three images were obtained from an alternate mouse (a total of 6 images from 2 mice brains). (**E**) Immunostaining of MAP2 in the cerebral cortex and hippocampus of 2-month-old mice. Insets show an enlargement of the micronuclei in the boxed area. Arrows indicate micronuclei. Scale bar: 50 μm. (**F**) Immunostaining of GFAP in the cerebral cortex and hippocampus of 2-month-old mice. Insets show an enlargement of the micronuclei in the boxed area. Arrows indicate micronuclei. Scale bar: 50 μm. Adobe Creative Could CC (Photoshop 22.1.0 and Illustrator 25.0.1) (https://www.adobe.com/) and FIJI Image J 2.1.0/1.53c (https://imagej.nih.gov/ij/) were used for all image processing in Fig. 1.

Because the micronuclei emerge in various tissues in response to genotoxic stimulation^8^, we investigated whether the micronuclei are observed in the wild-type tissues, such as the heart, skeletal muscle, testis, kidney, spleen, and thymus. We found that some micronuclei exist in these tissues in 2-month-old wild-type mice (Fig. 1A and B). The number of micronuclei in the spleen and thymus was smaller than expected, despite the fact that these cells are highly proliferative. Also, only a few small nuclei were observed in the heart and skeletal muscle, which are composed of non-dividing cells. These data imply that micronuclei are generated in each tissue to a greater or lesser extent. Next, we observed whether the brains possess micronuclei. Since neurons are not dividing cells, we speculated that few micronuclei exist in the cerebral parenchyma. Interestingly, several micronuclei were observed in the cerebral cortex and hippocampus, although they were small in number (Fig. 1C and D). Because the brain is composed of both neurons and glia, it is likely that glial cells have micronuclei in the cerebral parenchyma. To investigate this idea, we observed both neurons and astrocytes in the cerebral cortex and hippocampus. Micronuclei exist in both MAP2^+^ neurons and GFAP^+^ astrocytes (Fig. 1E and F), indicating that micronuclei appear in the brain ubiquitously. To summarize these observations, micronuclei exist in not only tumor tissues but also in various tissues of wild-type mice.

### Developing the micronuclei analysis tool using the MATLAB-based program

**Figure 2:**
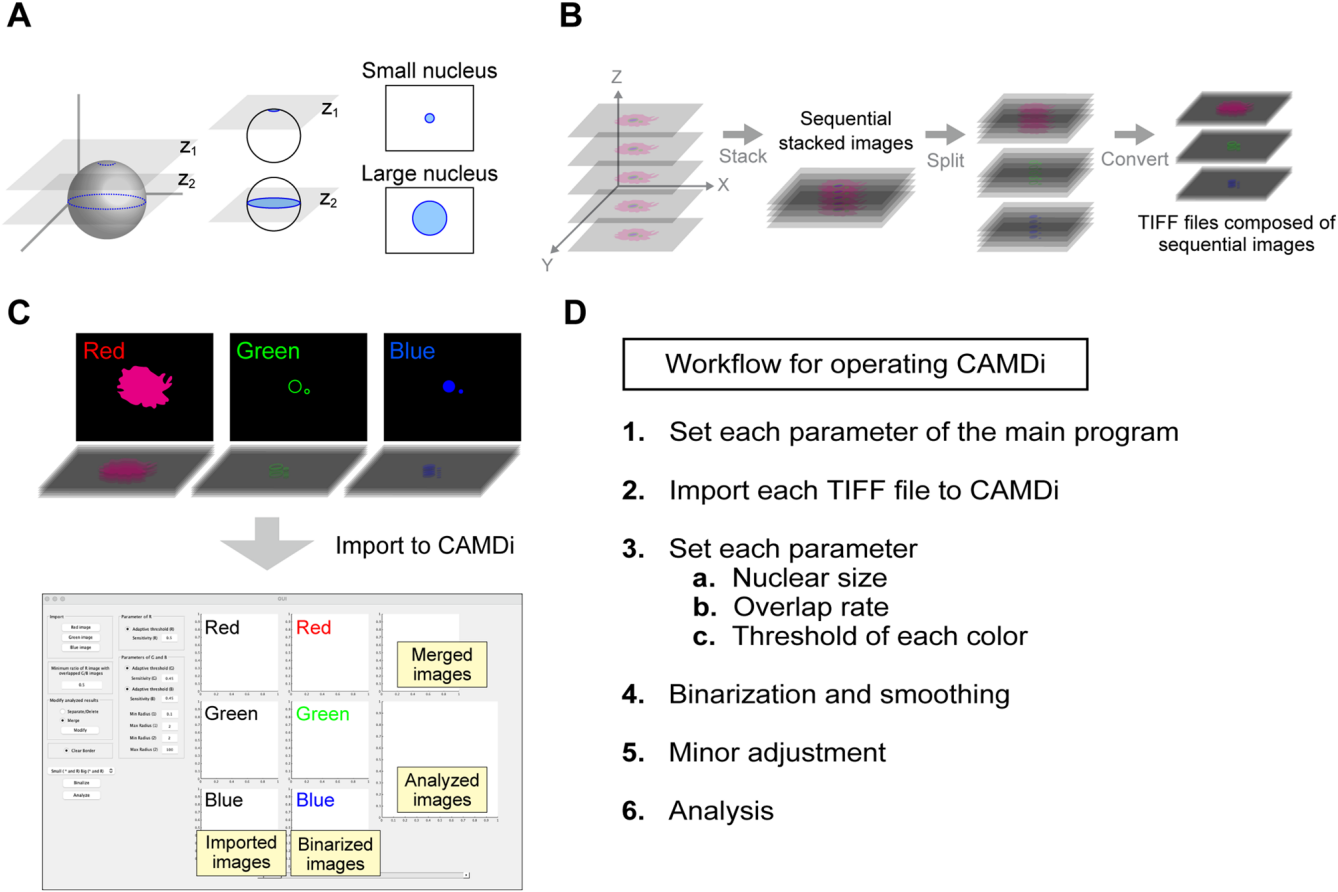
Developing the micronuclei analysis tool using the MATLAB-based program. (**A**) Schematic illustration of analyzing large and small nuclei. Nuclear size is altered if the images are taken from the different sections (Z_1_ and Z_2_). Z_1_: an upper section, Z_2_: a middle section. (**B**) Images need to be adjusted to a suitable format before importing into the CAMDi. Images should be a single or sequential TIFF file. Images are divided into three different colors (blue, green, and red). (**C**) Image processing step for importing data into CAMDi and representative window. Illustrations indicate cell body (red), nuclear envelope (green), and chromatin (blue). (**D**) Image processing workflow for quantification of micronuclei. Adobe Creative Could CC (Illustrator 25.0.1) (https://www.adobe.com/) was used for all illustration preparation in Fig. 2.

Since the micronucleus is defined by several parameters, such as nuclear components and size, the precise quantification of micronuclei is challenging. Several methods to quantify micronuclei have been reported to date^9,23–26^. The most frequently used method involves counting the small nuclei by analyzing the nuclear size as the index. This approach cannot, however, exclude the possibility that the regular nucleus is counted as a micronucleus when the edge of the nuclear section is identified using a confocal laser microscope (Fig. 2A). To address this, we developed a micronuclei analysis program, CAMDi, which quantifies the micronucleus precisely using MATLAB software. The conspicuous advantage of this CAMDi is that it can distinguish between micronuclei and regular nuclei. Sequential images with positional information are acquired using a confocal laser microscope. These images are stacked to a sequential image, divided into each channel, converted into a single TIFF file containing three-dimensional information, and imported into CAMDi. Next, the number of micronuclei is analyzed based on three-dimensional image information (Fig. 2B and C). CAMDi can also be utilized for not only three-dimensional but also two-dimensional images by setting the initial parameters. Furthermore, the CAMDi can define the micronuclear characteristic analyzed by nuclear components and cell-type specificity via co-staining with specific markers. The overall workflow for operating the CAMDi is illustrated in Fig. 2D.

### Processing flow of the MATLAB-based program, CAMDi

First, the initial parameters in the main program have to be defined. Information on each image, such as two-dimensional or three-dimensional, is inputted into CAMDi. Then, the parameter for the voxels, smoothing, and the radius of a nucleus is set in the "main" program through MATLAB windows (Table 1A). Next, the values to adjust the noise reduction in the smoothing process are defined to convert the binarized images accurately (Table 1B). Distinct nuclei are divided using the ellipse fitting functions (Table 1C). The ellipse fitting is usually an optional function to detect main nuclei.

**Table 1:** Setting initial parameters of MATLAB program.

**A. Set basic parameters of the main program (file: main.m) in CAMDi**	
prm.dim_cell_analyze = X;	Cell analyze (X); two-dimensional image, X-> 2; three-dimentional image, X-> 3
prm.dim_cell_radius = X;	Radius (X); Calculate from maximum area, X-> 2; Calculated from maximum volumn, X-> 3
prm.VoxelSpacing = [ X Y Z ];	Set scale (X, Y, Y); Distance in voxels (unit of length:1 μm)
prm.flag_insertNumber_outputImage = X;	Numbers (X); Show numbers in the binalized image, X-> 1, Don’t show numbers, X-> 0
prm.r_area_min = X;	Radius (X); Minimun radius of nucleus (R), unit [μm]
prm.r_area_max = X;	Radius (X); Maximum radius of nucleus (R), unit [μm]
prm3.rate_cover_r_min = X;	Overlap rate (X); Overlap rate between R and G/B, Regular value, X-> 0.5
**B. Noise reduction parameters of the main program, each color (file: main.m) in CAMDi**	
prm.smoothing.degreeOfSmoothing = X;	Maximum variance of pixel values in the smoothing area (X); Smoothing (R) off, X-> 0
prm.smoothing.spatialSigma = X;	Standard deviation of spatial radius in the smoothing area (X); Smoothing (R) off, X-> 0
prm.morphology.w1 = X;	Molphology scaling size for the area reconnection (X); Don’t reconnection (R) , X-> 0
prm.morphology.w2 = X;	Molphology scaling size for the noize reduction (X); Don’t noise reduction (R) , X-> 0
prm2.smoothing.degreeOfSmoothing = X;	Maximum variance of pixel values in the smoothing area (X); Smoothing (G) off, X-> 0
prm2.smoothing.spatialSigma = X;	Standard deviation of spatial radius in the smoothing area (X); Smoothing (G) off, X-> 0
prm2.morphology.w1 = X;	Molphology scaling size for the area reconnection (X); Don’t reconnection (G), X-> 0
prm2.morphology.w2 = X;	Molphology scaling size for the noize reduction (X); Don’t noise reduction (G), X-> 0
prm22.smoothing.degreeOfSmoothing = X;	Maximum variance of pixel values in the smoothing area (X); Smoothing (B) off, X-> 0
prm22.smoothing.spatialSigma = X;	Standard deviation of spatial radius in the smoothing area (X); Smoothing (B) off, X-> 0
prm22.morphology.w1 = X;	Molphology scaling size for the area reconnection (X); Don’t reconnection (B), X-> 0
prm22.morphology.w2 = X;	Molphology scaling size for the noize reduction (X); Don’t noise reduction (B), X-> 0
**C. Set ellipse fitting parameters of the main program, each color (file: main.m) in CAMDi**	
prm.FittingEllipse = X;	Maximum ratio of major/minor axis (X); The major/minor axis (R), off, X-> 0, circle X-> 1, ellipse- > > 1
prm2.FittingEllipse = X;	Maximum ratio of major/minor axis (X); The major/minor axis (G), off, X-> 0, circle X-> 1, ellipse- > > 1
prm22.FittingEllipse = X;	Maximum ratio of major/minor axis (X); The major/minor axis (B), off, X-> 0, circle X-> 1, ellipse- > > 1
prm22.FittingEllipse_NdivAng = X;	Division number of rotation (X); The turning angle = 180/FittingEllipse_NdivAng), Regular value, X-> 30

**Figure 3:**
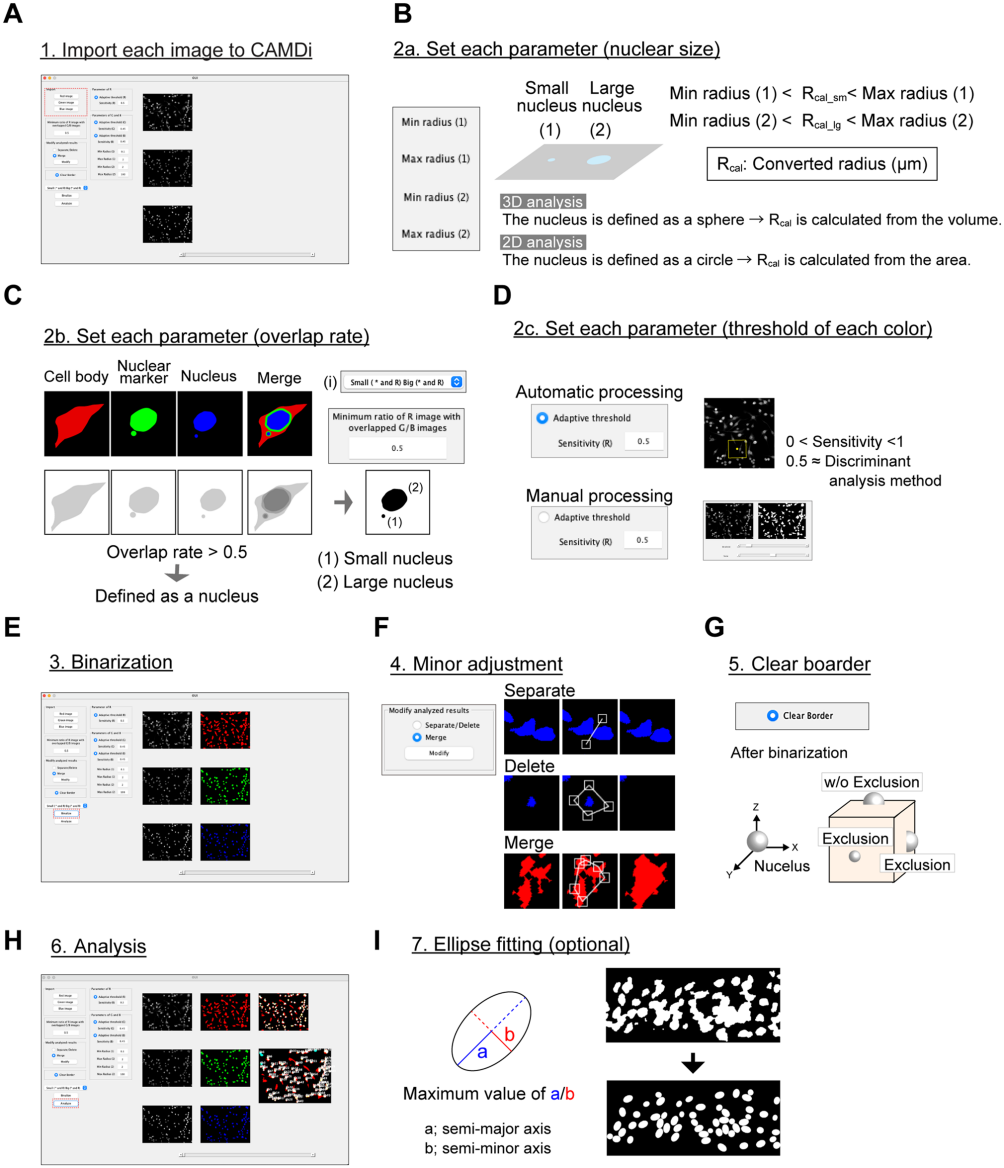
Processing flow of the MATLAB-based program, CAMDi. (**A**) The TIFF images are imported into CAMDi. (**B**) Definition of the size of the nucleus, (1) small nucleus, and (2) regular nucleus. (**C**) Definition of the overlapped rate: the default value is 0.5. Both overlapped areas and non-overlapped areas can be selected using window (i). (**D**) Definition of the threshold of signals. The discriminant analysis method is applied when automatic processing is selected. (**E**) Original images are converted into binarized images. (**F**) Merged or divided signals in the binarized images are corrected. (**G**) Signals on the border can be eliminated using this function. (**H**) Each data can be acquired after analysis. The result data are exported as csv files. (**I**) High-density signals are effectively separated using the ellipse fitting function. This function can only be used on the Microsoft Windows platform to date. Adobe Creative Could CC (Photoshop 22.1.0 and Illustrator 25.0.1) (https://www.adobe.com/) was used for all image processing and illustration preparation in Fig. 3.

Because the CAMDi program requires the TIFF images, it is necessary to convert the single or sequential images into TIFF images via the proper applications. Each image is imported through the "Import" windows (Fig. 3A). After importing the TIFF files, each parameter should be set up. CAMDi can distinguish between a small nucleus (micronucleus) and a large nucleus (regular nucleus in each cell). First, the radius of each nucleus is defined. The R_cal_ is a converted radius, which is calculated from the sequential images through the "main" program. In the case of three-dimensional analysis, R_cal_ is calculated from the volume after assuming that the nucleus is a sphere, whereas R_cal_ is calculated from the area after assuming that the nucleus is a circle in the two-dimensional analysis. Each parameter is defined as follows. Min. radius (1)-> minimum radius of micronucleus, Max. radius (1)—> maximum radius of micronucleus, Min. radius (2)-> minimum radius of nucleus, Max. radius (2)-> maximum radius of nucleus (Fig. 3B). To determine the characteristics of micronuclei, the rate of overlap area has to be calculated through the CAMDi windows or the "main" program. The default setting is 0.5, which represents 50% of the red area overlapped with the green or blue area. If a measurement value is higher than a set value, the nucleus is defined as an objective nucleus. Using the pull-down window (i) in Fig. 3C, non-overlapped nuclei with the red areas can also be counted (Fig. 3C). The fluorescence intensities of each color (red, green, and blue) are adjusted. This flow is implicated in the process of converting the binary image. CAMDi has two processes: the first is automatic processing, and the second is manual processing. In automatic processing, the value of sensitivity is adjusted. The sensitivity is utilized by a discriminant analysis method when the images are converted into binary images. In this step, the discriminant analysis is adaptively performed for each local area rather than for the whole image area. The window for manual processing emerges when the mark of "Adaptive threshold" is excluded. The threshold can be determined by altering the image frames (Fig. 3D). After setting each parameter, the images are converted into binary images. The binary images should be checked by eye to ensure they do not contain errors. Before the binarization, the images are executed by smoothing processing based on the bilateral filter. The bilateral filter is a smoothing filter that reduces noises in conjunction with preserving a part of the edges (Fig. 3E). Using the function of "modify analyzed results", each binarized image is corrected to have proper data, such as separation, deletion, and merge (Fig. 3F). The nuclei localized on the boundary of the x–z or y–z plane can be excluded after binarization when the "Clear border" box is checked. The nuclei on the x–y plane are not excluded (Fig. 3G). Finally, the data are analyzed using the binary images, and the number of micronuclei is quantified and exported as csv, txt, and tif files (Fig. 3H). In the high-density area, it is difficult to quantify the number of nuclei because each nucleus is fused after binarization. The "Ellipse fitting" function can convert the fused nuclei signals into clear ellipse shapes. Although the merged nucleus can be separated using the "modify analyzed results" window individually, the ellipse fitting functions can be defined as an individual nucleus automatically (Fig. 3I and Supplementary Fig. 1A to C).

### Inflammatory stimulations have little effect on micronuclei formation in neurons in vitro

**Figure 4:**
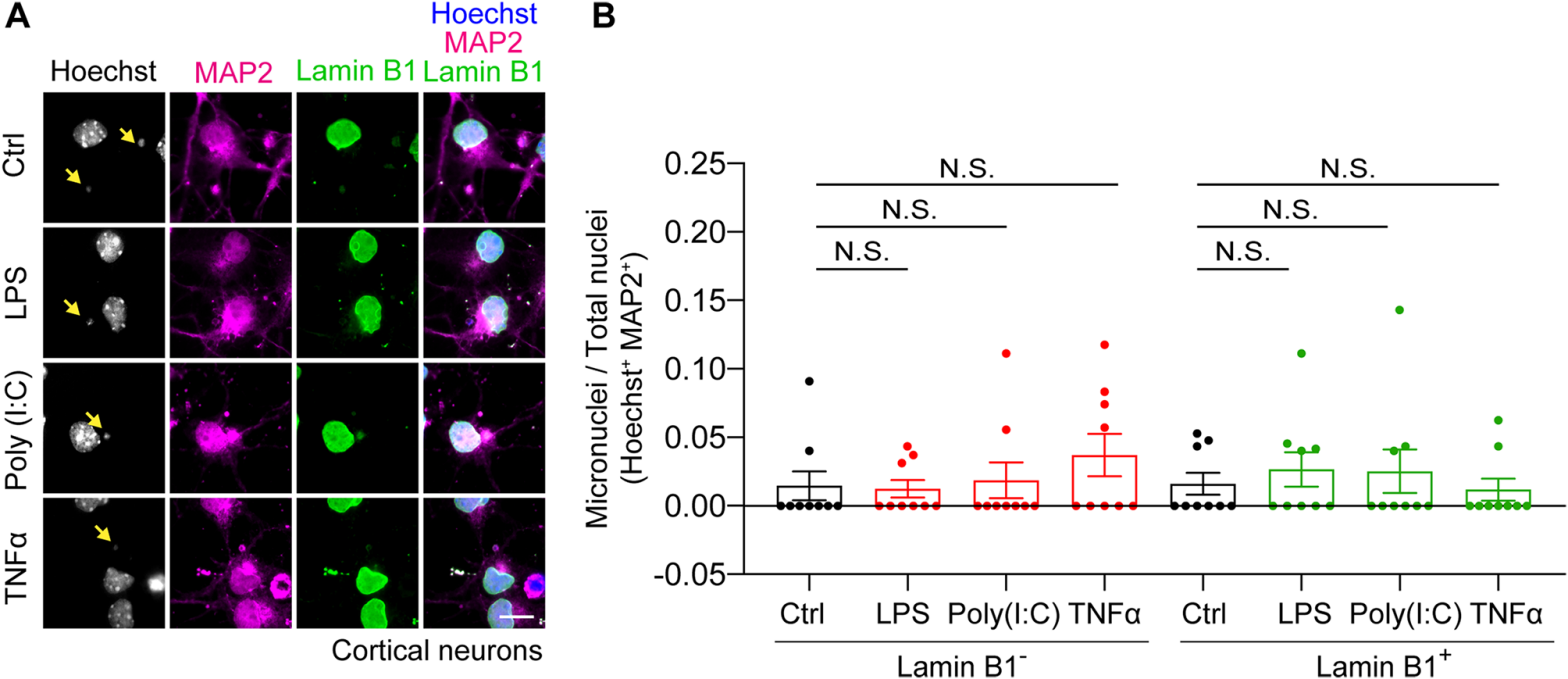
Inflammatory stimulations have little effect on micronuclei formation in neurons in vitro*.* (**A**) Primary neurons were plated at 1.0 × 10^6^ cells in 12-well plate after dissection, and incubated for 4 days. Immunostaining showing the micronuclei formation in the presence or absence of either 1.0 μg/ml LPS, 5.0 μg/ml Poly (I:C), or 50 ng/ml TNFα for 24 h. Arrows indicate micronuclei. Scale bar: 10 μm. (**B**) The graph shows the population of micronuclei positive neurons. Left columns: Lamin B1^−^ micronuclei, Right columns: Lamin B1^+^ micronuclei. The small nucleus (less than 2.0 μm) quantified by CMADi is defined as a micronucleus. n = 9 field; mean ± standard error of the mean (SEM), N.S. not significant by one-way analysis of variance (ANOVA) Tukey–Kramer statical tests. The data were reproduced in two independent experiments. Adobe Creative Could CC (Photoshop 22.1.0 and Illustrator 25.0.1) (https://www.adobe.com/) was used for all image processing in Fig. 4.

The mechanisms that underlie the micronuclei formation in the brain remain unknown. Using the two-dimensional mode of CAMDi program (prm.dim_cell_analyze = 2, prm.dim_cell_radius = 2), we investigated whether micronuclei can be analyzed in primary neurons. We determined that micronuclei exist even in primary neurons. Interestingly, the micronuclei were occasionally observed not only in the cell soma but also in the proximal dendrites and were coated by MAP2, which is a binding protein of the dendritic microtubules (Fig. 4A). These data suggest that the micronuclei are produced despite their small number and that they redistribute from the soma to the base of processes. Next, to investigate the link between neuroinflammation and neuronal micronuclei, we stimulated primary neurons with 1.0 μg/ml LPS, 5.0 μg/ml poly (I:C), and 50 ng/ml TNFα. Although the expression level of Toll-like receptors and TNF receptors in neurons is not high, neurons respond to these stimulations^27,28^. However, treatments with these stimulations did not significantly increase the number of micronuclei with or without the nuclear envelope in this system, suggesting that these stimulations have little effect on micronuclei formation directly (Fig. 4A and B).

### Inflammatory stimulation promotes micronuclei formation in vivo

**Figure 5:**
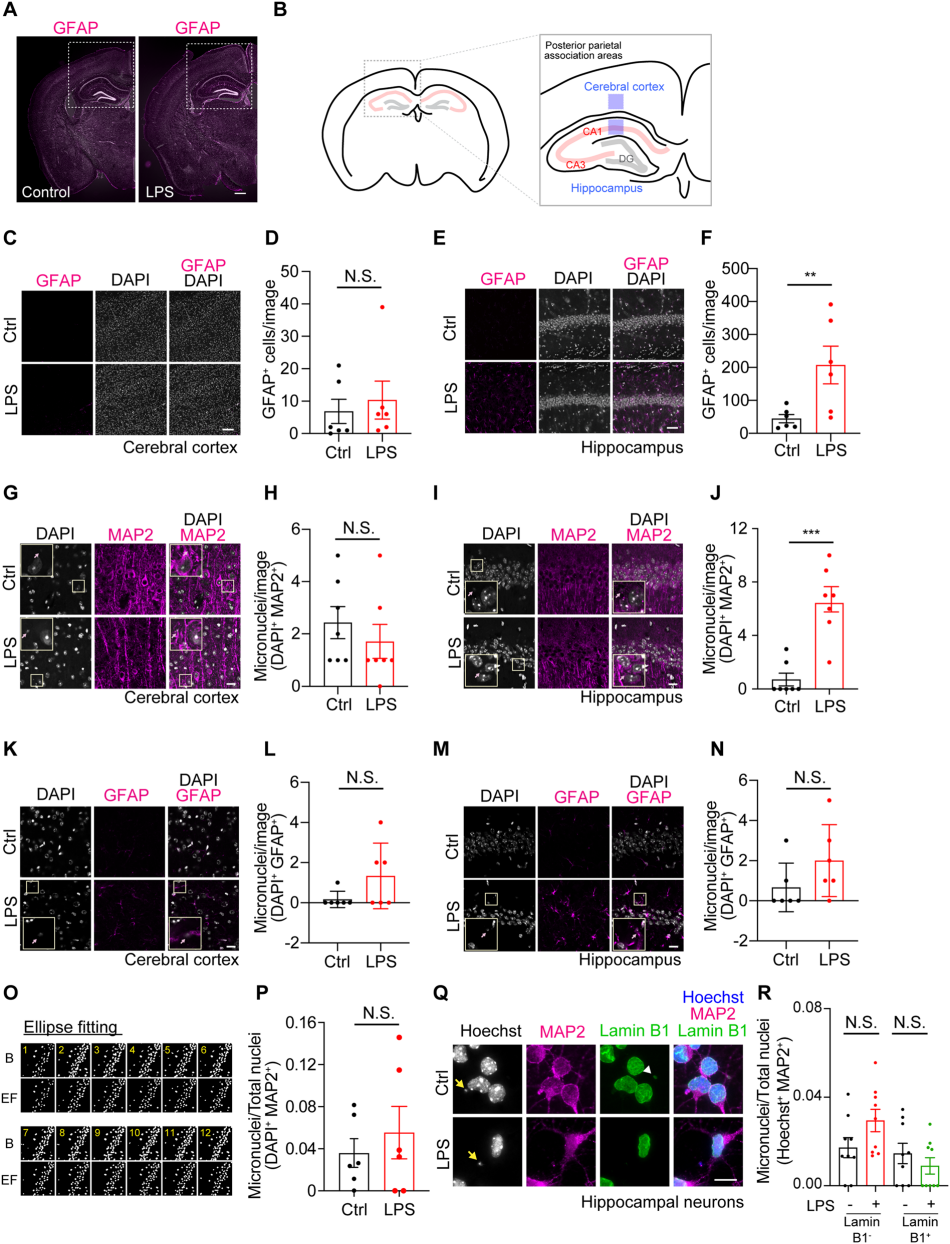
Inflammatory stimulation promotes micronuclei formation in vivo. (**A**) Immunostaining of GFAP in the whole brain of 2-month-old mice with or without 1.0 μg/kg LPS for 24 h. Insets indicate the quantified areas. Scale bar: 500 μm. (**B**) The detailed illustrations in the cerebral cortex and hippocampus indicate the quantified area in Fig. 5C to 5P. (**C**) Immunostaining of GFAP in the cerebral cortex of 2-month-old mice with or without 1.0 μg/kg LPS for 24 h. Scale bar: 50 μm. (**D**) The graph shows the population of GFAP-positive astrocytes. n = 6, 1.02 × 10^5^ μm^2^/image; 6 images obtained from 2 independent mice brains; mean ± SEM, N.S. not significant by Student's *t*-test. (**E**) Immunostaining of GFAP in the hippocampus of 2-month-old mice with or without 1.0 μg/kg LPS for 24 h. Scale bar: 50 μm. (**F**) The graph shows the population of GFAP-positive astrocytes. n = 6, 1.02 × 10^5^ μm^2^/image, 6 images obtained from 2 independent mice brains; mean ± SEM, **p < 0.01 by Student's *t*-test. (**G**) Immunostaining of MAP2 showing micronuclei in the cerebral cortex of 2-month-old mice with or without 1.0 μg/kg LPS for 24 h. Arrows indicate micronuclei. Scale bar: 20 μm. (**H**) The graph shows the population of micronuclei. The small nucleus (less than 2.0 μm) quantified by CAMDi was defined as a micronucleus. n = 7, 2.56 × 10^4^ μm^2^/image, 7 images obtained from 2 independent mice brains; mean ± SEM, N.S. not significant by Student's *t*-test. (**I**) Immunostaining of MAP2 showing the micronuclei in the hippocampus of 2-month-old mice with or without 1.0 μg/kg LPS for 24 h. Arrows indicate micronuclei. Scale bar: 20 μm. (**J**) The graph shows the population of micronuclei in the CA1 region. The small nucleus (less than 2.0 μm) quantified by CMADi was defined as a micronucleus. n = 7, 2.56 × 10^4^ μm^2^/image, 7 images obtained from 2 independent mice brains; mean ± SEM, ***p < 0.005 by Student's *t*-test. (**K**) Immunostaining of GFAP showing micronuclei in the cerebral cortex of 2-month-old mice with or without 1.0 μg/kg LPS for 24 h. Arrows indicate micronuclei. Scale bar: 20 μm. (**L**) The graph shows the population of micronuclei. The small nucleus (less than 2.0 μm) quantified by CMADi was defined as a micronucleus. n = 6, 2.56 × 10^4^ μm^2^/image, 6 images obtained from 2 independent mice brains; mean ± SEM, N.S. not significant by Student's *t*-test. (**M**) Immunostaining of GFAP showing the micronuclei in the hippocampus of 2-month-old mice with or without 1.0 μg/kg LPS for 24 h. Arrows indicate micronuclei. Scale bar: 20 μm. (**N**) The graph shows the population of micronuclei in the CA1 region. The small nucleus (less than 2.0 μm) quantified by CMADi was defined as a micronucleus. n = 6, 2.56 × 10^4^ μm^2^/image, 6 images obtained from 2 independent mice brain; mean ± SEM, N.S. not significant by Student's *t*-test. (**O**) Representative sequential images in the CA1 region before and after operating the ellipse fitting. B: binarized images before operating ellipse fitting, EF: converted images after ellipse fitting. (**P**) The graph shows the population of micronuclei. 6 images obtained from 2 independent mice brains; mean ± SEM, N.S. not significant by Student's *t*-test. (**Q**) The primary hippocampal neurons were plated at 1.0 × 10^6^ cells in 12-well plate after dissection and incubated for 4 days. Immunostaining showing the micronuclei formation in the presence or absence of 1.0 μg/ml LPS for 24 h. Arrows indicate micronuclei. Micronuclei without chromatin are occasionally observed (white arrowhead). Scale bar: 10 μm. (**R**) The graph shows the population of micronuclei-positive neurons. The small nucleus (less than 2.0 μm) quantified by CAMDi was defined as a micronucleus. n = 9 field; mean ± SEM, N.S. not significant by one-way ANOVA Tukey–Kramer statical tests. Data were reproduced in two independent experiments. Adobe Creative Could CC (Photoshop 22.1.0 and Illustrator 25.0.1) (https://www.adobe.com/) and FIJI Image J 2.1.0/1.53c (https://imagej.nih.gov/ij/) were used for all image processing in Fig. 5.

It is well known that most cells in the brain are glia, such as astrocytes. Against this background, we tested if glia activated by inflammatory stimulation influences micronuclei formation in the cerebral cortex and hippocampus (Fig. 5A and B). To investigate these regions, we administrated 1.0 μg/kg LPS into 8-week-old mice by intraperitoneal (i.p.) injection and analyzed the brain sections by immunohistochemistry. The administration of LPS had a small effect on the activation of astrocytes in the cerebral cortex under this condition (Fig. 5C and D). On the other hand, this stimulation clearly increased the number of reactive astrocytes in the hippocampus (Fig. 5E and F), suggesting that LPS injection enhances neuroinflammation in a region-specific manner. Next, we examined whether LPS administration increases the number of neuronal micronuclei in the brain. To determine this, we quantified the number of micronuclei using the three-dimensional mode of CAMDi (prm.dim_cell_analyze = 3, prm.dim_cell_radius = 2) after 24 h of LPS injections. Since micronuclei are frequently localized in the dendrites and soma (see Fig. 4A), we focused on the MAP2 positive regions. First, we observed the deep layer of the posterior parietal association area in the cerebral cortex. The LPS i.p. injection did not significantly increase micronuclei formation in the MAP2^+^ neurons of the cerebral cortex (Fig. 5G and H). Next, we observed the micronuclei in the hippocampus since the number of reactive astrocytes was increased after LPS injection. To examine this, we focused on the CA1 region (see Fig. 5B). Consistent with the results of GFAP immunostaining, the number of micronuclei in MAP2^+^ neurons was slightly increased in the hippocampus, indicating that the micronuclei formation induced by neuroinflammation is region-specific (Fig. 5I and J). Moreover, we investigated whether inflammatory stimulation induces astrocytic micronuclei formation in these areas. The number of astrocytic micronuclei was moderately but not significantly increased in both the cerebral cortex and hippocampus (Fig. 5K to N), indicating that LPS-induced micronuclei formation preferentially occurs in hippocampal neurons. Because the neuronal micronuclei are also localized in the cell soma, although few in number, we examined whether micronuclei were increased in the pyramidal layer of the CA1 region (Fig. 5O and P). To do this, we used the ellipse fitting functions to count the large nuclei because it is extremely difficult to analyze them with three-dimensional information due to their high density. The number of micronuclei in the soma was not increased following LPS stimulation, indicating that neuroinflammatory stimulation induces neuronal micronuclei in the hippocampus. Finally, we examined whether inflammatory stimulation promotes micronuclei formation in hippocampal neurons directly. To examine this, we treated primary hippocampal neurons with LPS and quantified the micronuclei. As a result, we found that LPS stimulation had little effect on micronuclei formation in primary hippocampal neurons. Intriguingly, the number of micronuclei with nuclear envelopes is slightly decreased independently of LPS stimulation (Fig. 5Q and R). These data suggest that LPS-induced neuronal micronuclei formation occurs in a non-cell-autonomous manner in the hippocampus region. Also, the retention time of the nuclear envelope in hippocampal neurons may be shorter than that in cortical neurons. As the nuclear envelope sequesters chromatin from cytoplasmic protein such as cGAS, the retention time of the nuclear envelope plays an important role in regulating the positive feedback of neuroinflammation in the hippocampal region. Taken together, these data suggest that micronucleus is a potential mediator for amplifying the neuroinflammatory response in the brain in a non-cell-autonomous event.

## Discussion

**Figure 6:**
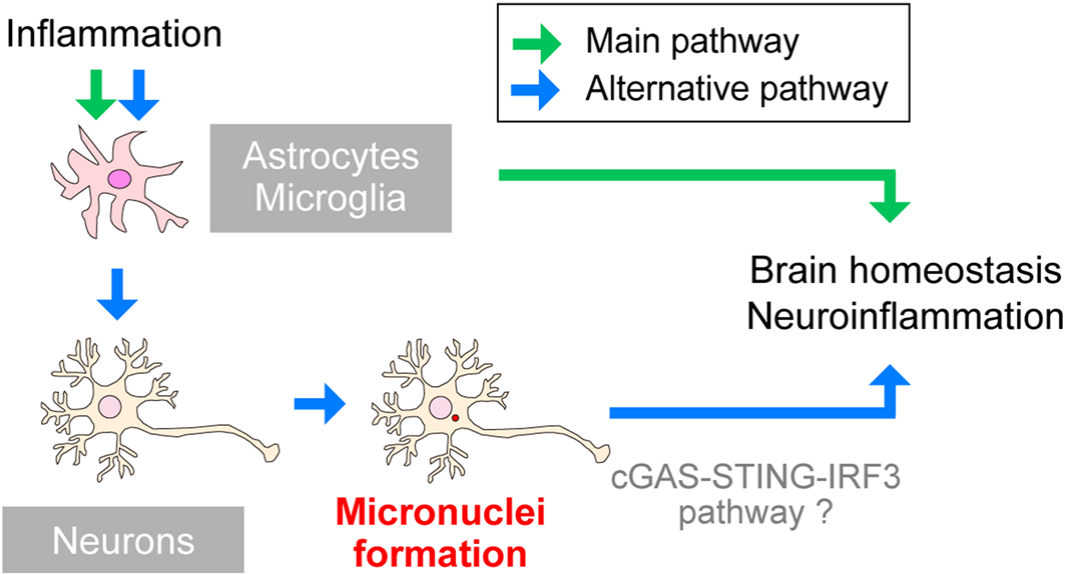
A model describing the pathways linking neuroinflammation to micronuclei formation. Inflammatory stimulations promote neuroinflammation via activating glia. Furthermore, inflammation produces micronuclei in neurons. Since micronuclei can activate the cGAS-STING pathway, micronuclei induced by the inflammatory response may act as a positive feedback pathway. Adobe Creative Could CC (Illustrator 25.0.1) (https://www.adobe.com/) and Apple Keynote 11.0.1 (https://www.apple.com/) were used for all illustration preparation in Fig. 6.

In this study, we developed a MATLAB-based program, CAMDi, for quantifying micronuclei. CAMDi is capable of analyzing the three-dimensional images and discriminating micronuclei from nuclei. This program can also detect the nucleus even in high-density areas. Using CAMDi, we revealed that neuroinflammation promotes neuronal micronuclei formation in the brain. We found that the inflammatory stimulation is not sufficient for inducing micronuclei in primary neurons. On the other hand, the neuronal micronuclei are increased following LPS administration in vivo, implying that the formation of neuronal micronuclei is not a cell-autonomous event. Our findings demonstrate that micronuclei are amplifiers for the inflammatory cascade in the brain (Fig. 6).

CAMDi provides a robust analysis of three-dimensional Z-stack images. In the micronuclei quantification to date, it is difficult to distinguish between the regular nucleus and the micronucleus using tissue images taken by a confocal laser microscope (see Fig. 2A). CAMDi can integrate the Z-stack information, reconstitute the nucleus stereographically in silico, and distinguish the micronuclei from the regular nuclei. Each threshold, such as size and Z-stack intervals, needs to be determined before analyzing. CAMDi provides robust and reproducible results and avoids person-to-person variability. This program is also useful when the cell density in the region is high (e.g., in the stratum pyramidale in the hippocampus). CAMDi can elicit each nucleus using ellipse fitting functions and analyze the suitable size of nuclei from Z-stack images, showing an accurate number of both nuclei and micronuclei. Previously, several groups have reported a high-throughput screening system for quantifying micronuclei in culture cells^9,23–26^. Although the precise quantification methods in vivo have not been developed, investigating micronuclei is crucial to understanding the pathological condition in each tissue. CAMDi enables us to evaluate the precise size of micronuclei via Z-stack sequential images. Additional staining, such as Lamin B1, helps us to understand the micronuclei conditions and to discriminate the micronucleus from artificial signals. Thus, CAMDi is a powerful application that is capable of acquiring accurate information from each tissue in physiological and pathological conditions.

Using CAMDi, we found that neuroinflammation increases the number of micronuclei in a region-specific manner. Recently, it has been reported that micronuclei enhance the inflammatory response in cancer cells via the innate immune system, the cGAS-STING pathway. The nuclear envelope is disrupted after forming micronuclei and the double-strand DNA is exposed to the cytoplasm. This cytoplasmic DNA activates cGAS, which synthesizes cGAMP, leading to the activation of STING on the endoplasmic reticulum (ER)^29^. Thus, it is likely that the micronuclei regulate the inflammatory response. On the other hand, the mechanisms that underlie neuroinflammation-induced micronuclei formation have not yet been elucidated. To date, it has been demonstrated that nuclear LC3/Atg8 regulates micronuclei formation in response to oncogenic stress, and this is followed by degrading the nuclear lamina^14^. During this process, the oncogenic stress, such as Ras activation, promotes this cascade. In contrast, inhibition of the autophagic pathway leads to accumulation of micronuclei in tumor cells. Although the precise mechanisms of how the oncogenic stress and the autophagic pathway regulates micronuclei formation have not been fully elucidated, it is plausible to consider that neuroinflammation affects these pathways.

Interestingly, we observed that micronuclei mainly increased not in the stratum pyramidale but in the stratum radiatum in the hippocampus region. Because micronuclei are frequently observed in the neuronal dendrites in primary neurons, they may redistribute from the soma to the processes in response to inflammatory stimulation. Another possibility is that micronuclei are produced in the early stage and are sustained in the neurons of the postnatal brain. Our preliminary data suggest that neurons can form micronuclei when they pass through the narrow region (Yano et al*.* in preparation). Because part of the hippocampus is a high-density region, the mechanical stress during migration could produce the micronuclei that maintain neuronal dendrites while avoiding their degradation. Why the micronuclei exist in the dendrites remains unknown. As the ER in neurons is also expandeds to the dendrites and spines^30,31^, micronuclei in the proximal dendrites could be crucial for activating the cGAS-STING cascade locally. Because the micronuclei and cGAS-STING pathway are involved in the neurodegeneration such as Alzheimer's disease (AD), Parkinson's disease (PD), and amyotrophic lateral sclerosis (ALS)^21,32,33^, the micronuclei-cGAS-STING pathway is a potential candidate linked to these disorders. It is important note that neuronal micronuclei are not induced by stimulation with LPS, TNFα, and poly (I:C) directly. On the other hand, the administration of LPS activates astrocytes and promotes neuronal micronuclei formation in the hippocampus region. As reactive astrocytes secret a variety of proteins under stress conditions^34^, several factors released from astrocytes may affect neuronal micronuclei formation.

Overall, we developed a MATLAB-based program to analyze micronuclei in the stereoscopic images in vivo. We believe that the CAMDi will serve as a powerful tool to answer the interesting remaining questions regarding cellular motility, inflammation, and chromosomal instability. Moreover, our finding may shed light on the unclarified mechanisms linking neuroinflammation to cognitive impairments and neurodegeneration^35,36^.

## Methods

### Materials and cell cultures

Anti-Lamin B1 (abcam, ab16048), anti-MAP2 (Merck Millipore, MAB378), and anti-GFAP (SIGMA, G-A-5) antibodies were used for immunocytochemistry and immunohistochemistry. Hoechst33342 was purchased from Thermo Fisher Scientific. DAPI was purchased from DOJINDO. Primary neurons were prepared from E13.5–14.5 Crl:CD1(ICR) mice as previously described^37^. Briefly, mouse cortical neurons were plated on coverslips coated with 0.01875% Poly (ethylenimine) solution (SIGMA) and cultured in Neurobasal medium (Thermo Fisher Scientific) containing B-27 supplement (Thermo Fisher Scientific) and 100 units penicillin/100 mg streptomycin (P/S) (Thermo Fisher Scientific). Neurons were maintained for 4 days at 37℃ with 5% CO_2_ and were then subjected to immunocytochemistry.

### Immunocytochemistry

Primary neurons (4 days in vitro) were stimulated by either 1.0 μg/ml LPS (SIGMA), 50 ng/ml TNFα (R&D Systems), or 5.0 μg/ml Polyinosinic-polycytidylic acid (Poly I:C) (SIGMA) for 24 h and were then subjected to immunocytochemistry. Primary neurons were fixed with 4% paraformaldehyde (PFA) in phosphate-buffered saline (PBS) for 10 min at room temperature. Neurons on coverslips were blocked in 0.4% Triton X-100 in blocking solution (3% bovine serum albumin [BSA] in PBS) for 30 min at room temperature and then incubated with primary antibodies (anti-MAP2 antibody [1:500] and anti-Lamin B1 antibody [1:500]) diluted in blocking solution for 2 h at room temperature. After washing with PBS, the cells were incubated with either anti-rabbit Alexa Fluor Plus 488 (1:500, Thermo Fisher Scientific) or anti-rabbit Dylight Fluorochome 488 (1:500, abcam) conjugated secondary antibodies and anti-mouse Dylight Fluorochrome 594 (1:500, abcam) conjugated secondary antibody diluted in blocking solution for 60 min at room temperature. Nuclei were stained with 10 μg/ml Hoechst 33,342. The coverslips were then mounted onto slides using FLUOROSHIELD Mounting Medium (ImmunoBioScience). Fluorescent images were obtained using a fluorescence microscope (BZ-9000, Keyence) with 40× (PlanApo 40×/0.95) and 100× (PlanApo VC 100×/1.4 Oil) objective and a charge-coupled device camera (Keyence). A proper wavelength was selected using fluorescence filter cubes;DAPI [Ex 360/40, DM 400, BA 460/50] (Keyence), GFP-B [Ex 470/40, DM 495, BA 535/50] (Keyence), mCherry-A [Ex FF01-562/40-25, Di FF593-Di02-25 × 36, Em FF01641/75-25](Opto-Line, Inc).

### Immunohistochemistry

The C57BL/6 mice (2 months old) were injected with 1.0 mg/kg LPS (SIGMA) for 24 h and were then anesthetized with isoflurane. The brains were perfused with 4% PFA-PBS and fixed with the same fixative solution overnight. After 30% sucrose-PBS infiltration, the samples were embedded in Tissue-Tek Optical Cutting Temperature (O.C.T.) compound (SAKURA) and sliced at 30 μm thickness using a cryostat (Leica Biosystems). The tissue sections were blocked for 1 h in 5% BSA in PBS and incubated with a primary antibody in 0.1% Triton X-100 in TBS (25 mM Tris–HCl [pH 7.5], 0.14 M NaCl) for either 1 day (anti-GFAP antibody) or 3 days (anti-MAP2 antibody) at 4 °C. Following the wash with TBST, the tissue sections were incubated with an anti-mouse Dylight Fluorochrome 594 (abcam) secondary antibody diluted 1/500 in TBST together with 1.0 μg/ml DAPI for 1 h at room temperature. The tissues were then mounted onto slides using VECTASHIELD Mounting Medium (Vector Laboratories). Tissue specimens were observed using a confocal laser scanning microscope (LSM700, Carl Zeiss) with 20× (Plan-Apochromat 20×/0.8 M27) and 40× (Plan-Apochromat 40×/1.3 Oil DIC M27) objective. The diode excitation lasers (Diode 405, Diode 488, and Diode 555) were operated and directed to a photomultiplier tube (LSM T-PMT, Carl Zeiss) through a series of band pass filters (Ch1:BP420-475 + BP500-610, Ch2:BP490-635, and Ch3:BP585-1000). The Z-stack images (interval: 1 μm per image) were acquired using ZEN software 2009 (Carl Zeiss). All of the animal experiments were conducted according to the university guidelines for animal care and use and arrive guidelines. This study was approved by the Animal Experiment Committee in the University of Tsukuba (the approval numbers:19-340, 20-438).

### Micronuclei imaging

The C57BL/6 mice (2 months old) were anesthetized with isoflurane. Each tissue (heart, skeletal muscle, kidney, spleen, thymus, testis, and brain) of mice was perfused with 4% PFA-PBS and fixed with the same solution overnight. After 30% sucrose-PBS infiltration, the samples were embedded in Tissue-Tek O.C.T. compound (SAKURA) and sliced at a 30 μm thickness. The tissue sections were then incubated at 10 μg/ml Hoechst/TBST (heart, skeletal muscle, kidney, spleen, thymus, testis) or 1.0 μg/ml DAPI/TBST (brain) for 2 h. Tissue specimens were observed using a confocal laser scanning fluorescence microscope (LSM700, Carl Zeiss).

### Micronuclei analysis

FIJI ImageJ software (NIH) and ZEN (Carl Zeiss) were used for converting each Z-stack image (interval: 1 μm per image, total thickness: 10–15 μm) to a single TIFF file. The data were imported into MATLAB software, CAMDi, which can analyze three distinct colors (e.g., blue: nucleus/micronucleus; green: the marker for nucleus/micronuclei; red: cell markers). Quantifying the marker, such as Lamin B1, provides us information on the status of the nuclear envelope and chromatin conditions. The imported data were conducted binarization and the threshold of each color was determined. After making the binary images, the merged areas were extracted according to the threshold and the number and area were quantified. The size of micronuclei has been reported to be less than one-third of main nuclei^38,39^. Because the major axis of the nucleus is approximately 12 to 15 μm in neurons and astrocytes, the maximum value of a semi-major axis was set up at 2.0 μm in CAMDi. All data were reproduced in at least two independent experiments.

### Statistical analysis

The statistical data were calculated by GraphPad Prism 8 (GraphPad Software), and compared by Student's *t*-test and one-way analysis of variance (ANOVA).

## Supplementary Information

Supplementary Information.

## Data Availability

The CAMDi software is available upon request: tsuruta.fuminori.fn@u.tsukuba.ac.jp.
