# Supplementary figures and images for: A MATLAB-based program for three-dimensional quantitative analysis of micronuclei reveals that neuroinflammation induces micronuclei formation in the brain
Source: Sci Rep. 2021 Sep 15;11:18360. doi: 10.1038/s41598-021-97640-6 (PMC8443747; doi:10.1038/s41598-021-97640-6)

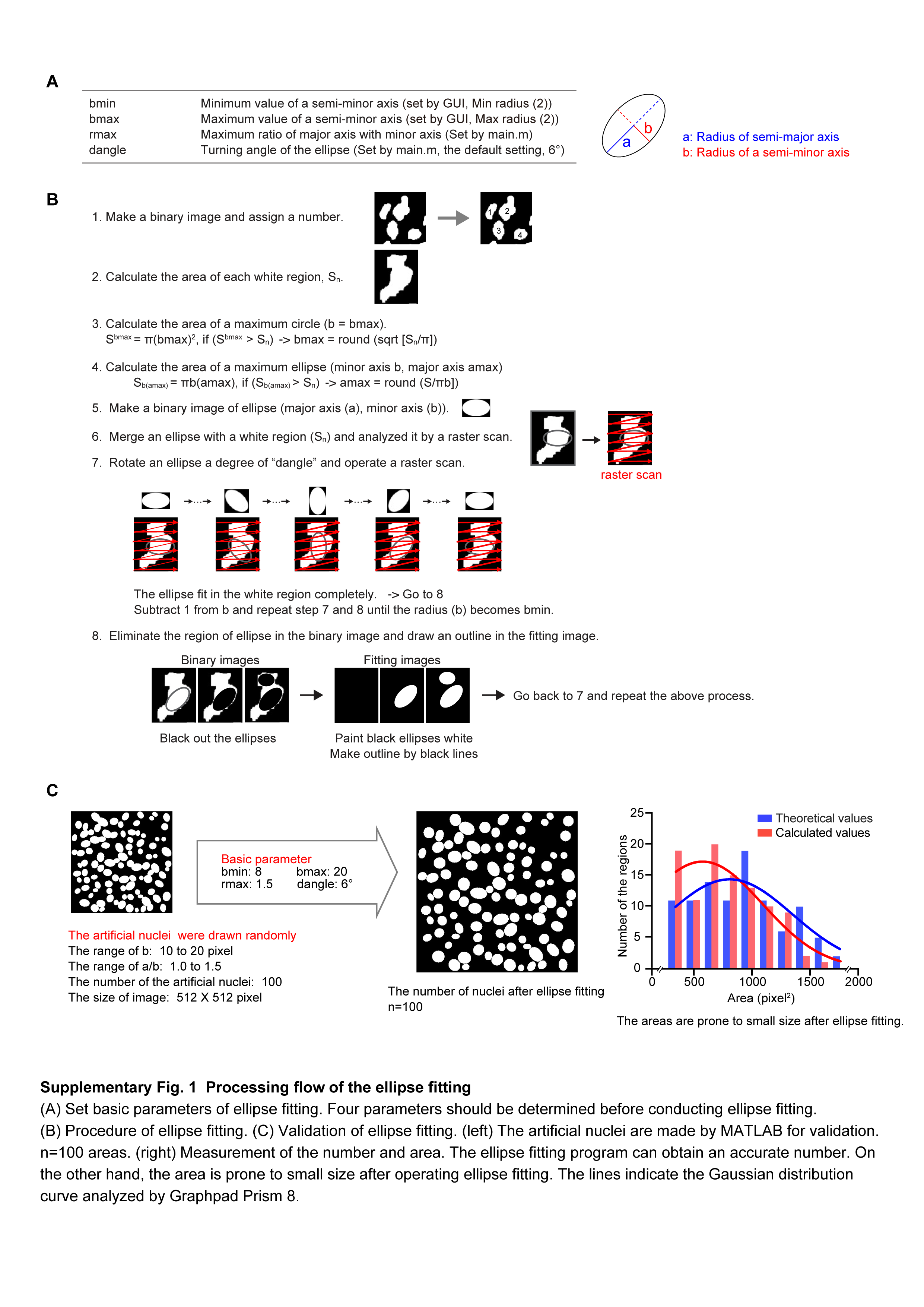

Supplement: Supplementary file 1 — Supplementary Information. [file 41598_2021_97640_MOESM1_ESM.tif]
